# AI overuse in education extending the technology acceptance model with Confucian cultural moderation

**DOI:** 10.3389/fpsyg.2026.1813693

**Published:** 2026-07-14

**Authors:** Jingyi Li, Lianyun Huang, Fumitaka Furuoka

**Affiliations:** 1 School of Finance and Tourism, Chongqing Vocational Institute of Engineering, Chongqing, China; 2 Modern Service Industry Development Center of Jingyang District, Deyang, Sichuan, China; 3 High-end Talent Service Center of Jingyang District, Deyang, Sichuan, China; 4 Asia-Europe Institute, University of Malaya, Kuala Lumpur, Malaysia

**Keywords:** AI technology overuse, Confucian values, habit, satisfaction, technology acceptance model

## Abstract

Artificial Intelligence (AI) assisted writing tools, such as ChatGPT have arisen rapidly and created a socio-technical phenomenon of fast technology adoption in higher education. Traditional models focus on early adoption but understanding the behavioral pathway that accounts for intensive AI overuse is critical. To summarize, this study enriched the Technology Acceptance Model (TAM) by developing a sequential mediation model: Perceptions (PU/PEOU) → Satisfaction → Habit → AI Overuse and investigated boundary conditions of this process regarding Confucian Cultural Values as moderator. By conducting the survey mixed-mode, from December 2024 to February 2025 data has been collected using a systematic random sampling of Chinese educators at six universities in Chongqing through both individual intercepting and online distribution. Direct, mediation and moderation effects were tested based on Partial Least Squares Structural Equation Modeling (PLS-SEM). Results confirm partial mediation: utility and ease perceptions drive user satisfaction, to the extent that it spurs behavioral automaticity (habit) behind high-intensity use the study characterizes AI Overuse as a relatively high force and goal-oriented behavioral outcome of technology acceleration, separating this from clinical addiction. As for the ability to moderate, Confucian Values firmly bolster the impact of Perceived Usefulness upon Satisfaction according to the power placed on collective welfare. Interestingly, these cultural effects are moderated when the variables Perceived Ease of Use (a cognitive barrier) and Habit (a psychological boundary) are applied. Theoretically, the current research offers a new lens through which accelerated technology adoption can be analysed and guides tailored digital sustainability interventions in Confucian models of education.

## 1 Introduction

Artificial intelligence (AI) has dramatically transformed writing practices in academia thanks, in large part, to the commercialization of AI-authored tools for writing, for example, ChatGPT and Grammarly or iFlyTek Smart Input. They make use of natural language processing, machine learning and big data analytics to enabling drafting, paraphrasing, summarising and feedback generation tasks (Novelli et al., 2024; Herrmann, 2023). In educational contexts, they have been harnessed by teachers to create materials for lessons, write feedback on assessments, improve academic communication and ease administrative written tasks (Chiu et al., 2023). AI writing assistance has emerged to be one of the most significant innovations shaping contemporary pedagogy, with promises of efficiency, linguistic accuracy, and cognitive offloading (Bearman et al., 2023). The adoption of AI-enhanced writing tools has been especially prominent in academic writing, where education professionals have increasingly turned to writing aid solutions that employ AI algorithms, propelled by growing demands on workloads and expectations regarding written output (Crompton and Burke, 2023) in the Chinese vocational and higher education sectors.

Nevertheless, the sudden normalisation of writing aids provided by AI has also raised public anxiety regarding over-reliance (Yankouskaya et al., 2025), particularly within academia in which writing is associated with professional authority and intellectual integrity (Wang et al., 2024). The framing of digital overuse through the lens of addiction (Bellis et al., 2021) is a common existing discourse although educators’ usage patterns of AI writing tools would not necessarily be considered pathological. For example, much of the so-called AI “overuse” occurs not as compulsive behavior, but from rational urges to seek out efficiency in work. AI is low cost to use, readily accessible, and its interface design is persuasive enough to support habit forming behavior (Thaler and Sunstein, 2009; Montag and Walla, 2016), which creates a thin line between productive augmentation and abuse dependency. Thus, this research frames the overuse of AI in writing not as a clinical disorder but rather as a socio-technical phenomenon that features permanent reliance on artificial text to complete basic writing tasks, making human authorship transition from active writer to passive overseer.

Understanding this trajectory requires a process-oriented perspective. The technology acceptance model (TAM) proposes that users have perceived usefulness (PU) and perceived ease of use (PEOU), the perceptions being user driven or initiation adoption. When applied to AI-based writing tools, PU could manifest as an oversensitivity to the clarity, speed or professionalism of the written output; while PEOU denotes greater integration of AI tools into everyday writing workflows. TAM covers early adoption but is weak in explanation after adoption (Obal, 2017). The research on post-acceptance behavior indicates that satisfaction triggers continued usage through positive reinforcement (Shahzad et al., 2024), and repeated engagement entails the formation of habits (Eyal, 2014). In writing contexts, educators with positive experiences of ongoing benefits from AI-assisted writing processes may move over time from optional assistance to default reliance on the machine, less engaged critically and reflectively, and thereafter lose some sense of agency in their composing. However, very little before have studies tested this satisfaction–habit route in AI-assisted writing.

Traditional culture is broadly recognized as a major systemic factor influencing individual choices to adopt new technologies (Jackson and Harris, 2003; Zhang et al., 2019). However, although information technologies can be technically useful and easy to use, people often only accept them if they match with stable traditional cultural values. Technologies that strive for efficiency alone and do not support these values might be seen with suspicion or as bad even when they are technically superior. Chinese traditional culture is deeply rooted in Confucian values (Wei-Ming, 1991) and further affects people’s behavior and decision-making. But though Confucianism calls for a cautious and responsible attitude about technological advance, technology should help us be human rather than the opposite (Muyskens et al., 2024) that could ironically motivate high use dramatization. Technology is not value-neutral and its benefits are expected to maintain basic community values like harmony (Zhu, 2020). The value of understanding satisfaction is determined by how collectivists put group needs before their own, which includes the focus on others that pervades Confucianism (Cho, 2011). Consequently, Chinese educators may feel compelled to utilize AI tools excessively if they believe such efficiency benefits the collective educational community, risking over-reliance and loss of control in the process. Using this cultural lens to look at AI in education shows that Confucian values may shape how people see AI technologies and how satisfied they are with them.

To address these gaps, this study proposes the AI Writing Overuse Model for Educators. Specifically, this study is guided by the following research questions (RQs):

*RQ1*: How do educators’ perceptions of AI writing tools (PU and PEOU) influence AI overuse through the sequential mediation of satisfaction and habit?

*RQ2*: How do Confucian value orientations moderate the relationships between technology perceptions, satisfaction, and habit formation?

It is worth clarifying that this study does not formally present null or alternative hypotheses alongside the directional hypotheses. This is consistent with the methodological conventions of PLS-SEM-based exploratory research (Hair et al., 2019; Hair et al., 2021), in which the objective is theory development and prediction rather than the classical null-hypothesis significance testing (NHST) framework. In PLS-SEM, bootstrap-derived confidence intervals and t-values are used to evaluate path significance, and the analytical logic is oriented toward estimating the magnitude and direction of relationships rather than rejecting null hypotheses in a confirmatory sense. Presenting formal null hypotheses (H0) would be more appropriate in a covariance-based SEM (CB-SEM) context designed for hypothesis falsification. Because the present study adopts an exploratory stance to extend TAM into a novel cultural and behavioral domain, directional research hypotheses grounded in theory are the appropriate form of proposition, as is standard practice in the educational technology and information systems literature that employs PLS-SEM (e.g., Ghasemy et al., 2020; Tarhini et al., 2017). The contribution is threefold. First, it reframes AI overuse as a non-pathological behavioral outcome arising from routine technology acceptance rather than addiction. Second, it extends TAM by incorporating post-adoption psychological mechanisms and socio-cultural moderation. Third, it provides theoretical and practical insight into educators’ increasing dependence on AI writing tools, informing policies for responsible and sustainable integration. To our knowledge, this is the first study to examine AI writing overuse among educators through a combined lens of technology acceptance, behavioral habit formation, and Confucian cultural ethics. The theoretical framework of this study is grounded in internationally recognised, high-impact scholarship. The core literature draws primarily from top-tier, internationally indexed journals, including MIS Quarterly, Journal of the Academy of Marketing Science, Higher Education, Computers and Education: Artificial Intelligence, British Journal of Educational Technology, International Journal of Educational Technology in Higher Education, Nature, Frontiers in Public Health, and European Business Review, among others. These sources span the leading outlets in information systems, educational technology, behavioral science, and social psychology, ensuring that the conceptual foundations and empirical precedents cited are both rigorous and globally recognized. Where regionally focused studies are included, they are selected specifically to capture the cultural and institutional context of Chinese higher education, which is directly relevant to the study’s research setting and cannot be adequately represented by Western-centric literature alone.

## 2 Understanding of AI overuse

Digital overuse (DO) is the result of an evolutionary socio-technical process that develops from intricate interrelations between human cognition, social pressure, and different technological advances. This DO is not simply a linear gain of screentime, it is instead the qualitative transformation of new modes and vices that power each temporal conduit. The phases each display the amplification of human cognitive predispositions through the introduction of new affordances, rewriting patterns of attention, social interaction, and, most recently cognition itself.

Digital Overuse 1.0 aligns with the early Internet and social media dawn. It is rooted in the interplay of human attentional biases and the natural affordances of new digital spaces. From an evolutionary perspective, humans are programmed to be attracted to new and conspicuous stimuli (and for good reasons in regard to survival in ancestral environmental conditions) (Anderson, 2013). Traditional digital platforms took advantage of this impulse with endless scrolling, autoplay and algorithmic feeds that incessantly prioritized new information, or surprise (Alter, 2018). These mechanisms caused a state of enduring attentional capture, and led to behavioral excess reflected by passive consumption. This was psychologically driven by attentional depletion and the exertion of cognitive control over time through absorbed, reward-seeking streams of splintered content (Kahneman, 2011). Such engagement was reinforced by multiple reinforcement schedules of the reward circuits of the brain, maintaining long and short-term use. Societally, this era characterized the monetization of attention: platforms earned income from advertisers who paid to reach users’ attention (Williams, 2018). As a result, DO 1.0 lay the groundwork for a feedback loop between human cognition and platform economics that turned attentional scarcity into a commodity.

With the rise of smartphones and mobile applications, Digital Overuse 2.0 transitioned from simply consumption to being constantly connected. The problem of excessive use emerged because societal cognition, particularly one that was sensitive to the validation and belonging of others within human environments, converged with affordances inherent in mobile technologies built on their ability to connect different people at pace (Vanden Abeele, 2021). The arrival of mobile notifications, instant messaging, and real-time social feeds introduced near-identical intermittent reinforcement patterns based on well-known operant conditioning paradigms (Skinner, 1965) which resulted in compulsive checking behavior and fear of missing out. Psychologically, DO 2.0 increased dependence by mingling reward dependency and anxiety-driven activities to create patterns of hyper-connectivity with silos of reduced autonomy. At the societal level, being accustomed to having a mini-computer with us all the time normalized immediacy as a social expectation and an expectation within our lives. It was now not only technologically possible, but a societally encouraged behavior, as we equated disconnection to inefficiency and dereliction. So, this stage is rooted in the institutionalisation of connectivity, where technological convenience became cultural necessity, and overuse transferred from individual behavior to social expectation.

Digital Overuse 3.0, or AI Overuse is a critical paradigm shift in the evolution of human–technology relations. Cognitive outsourcing, reasoning, problem-solving, and creative production delegated to intelligent systems (Wang et al., 2025), is at the heart of DO 3.0 Generative AI, automated tutoring and writing assistants demonstrate the capability to perform tasks that were previously considered as requiring explicit human cognition, allowing users to offload intellectual effort in search of efficiency (Jin and Uchida, 2024). This is driven by the cognitive economy, where people would rather avoid mental effort when technology can do it for us. Consequently, this form of dependence breeds competence displacement, a psychological mechanism that involves the decline in users’ perception of their own capability and self-efficacy which occurs when they become increasingly reliant on algorithmic output (Long and Magerko, 2020).

prior overuse affected attention and behavior, DO 3.0. Delegating cognitive effort to machines threatens self-efficacy, creativity and reflection. This leads to a capability-based dependency, wherein human sense-making and authorship become progressively displaced by the passive stewardship of machine-generated content. Societally, this phase would mirror the mainstreaming of cognitive delegation (Rahwan et al., 2019), where AI systems fold into work and school routines while establishing new productivity benchmarks. Tying into the work of Gui and Büchi (2021) who framed digital overuse as a social phenomenon rooted in ever developing digital technologies which improve their functionality through increasing societal acceptance, this phase expands the logic of overuse not only into attention and connectivity but toward cognition itself. The adoption curves from Internet to mobile computing and now artificially intelligent systems all follow a similar trajectory of functional expansion before social normalization. With AI being fully integrated into educational, professional, and creative contexts it reshapes not only how people use technology, but also how they think.

Thus, this study frames AI writing overreliance not as a clinical disorder but as a socio-technical phenomenon defined by excessive, repeated use of AI-generated text for mundane writing activities where human authorship de-emphasizes active generation and emphasizes passive supervision. In this context, excessive use of AI writing devices is the manifest realization of a long history trajectory spanning from attention-directed overuse to capability-based reliance, revealing an overall trend of cultural normalization of AI as an extension and replacement both in its role as active cognitive partner and diminished human creative agency. It emphasizes the importance of theorizing AI overuse not simply as use of this technology to excess, but as a change in how intellectual labor should be envisioned, in concert with our co-evolution with human cognition and social expectations balanced against technological affordances.

## 3 Theory and hypotheses

### 3.1 Technology acceptance model

Many information technologies have become well established, yet users often demonstrate reluctance or resistance toward their adoption (Al-Nuaimi and Al-Emran, 2021). Understanding such reluctance requires examining the psychological mechanisms that shape individuals’ technology-related perceptions and decisions. Davis (1986) established the TAM, which is grounded in the Theory of Reasoned Action (TRA) and focuses on both cognitive judgment and affective response when users form intentions to use technology. TAM provides a robust framework for analyzing predictors of technology acceptance (Al-Adwan et al., 2023) and has been extensively applied to explain new technology adoption across multiple domains (e.g., Davis, 1986, 1989; Tetik et al., 2024; Al-Adwan et al., 2023; Saif et al., 2024; Fussell and Truong, 2022).

In the classical TAM, PU and PEOU are established as antecedents of technology acceptance, typically measured by behavioral intention and actual use (Davis, 1989). Nevertheless, this study hypothesizes that PU and PEOU influence user satisfaction rather than acceptance. This shift reflects a move away from the concern of initial adoption toward the evaluation of post-adoption experiences, aligning with recent TAM extensions. Once users become comfortable with a technology, satisfaction may serve as a more meaningful and stable outcome than mere acceptance (Almulla, 2024).

In the educational context of AI-assisted writing, this study adopts TAM to examine the direct effects of PU and PEOU on educators’ satisfaction with AI writing tools. PU refers to the degree to which an individual believes that using an AI writing tool enhances their teaching or writing performance, while PEOU refers to the degree to which they perceive the tool as easy to use (Davis, 1989). Educators who perceive AI writing tools as both useful and effortless are more likely to experience satisfaction because such perceptions reinforce the belief that the tools improve instructional productivity and reduce workload. This satisfaction reflects a positive affective and evaluative reaction (Han and Sa, 2022; Legramante et al., 2023; Ruangkanjanases et al., 2024). Accordingly, the study proposes the following hypotheses:

*H1*: Perceived Usefulness (PU) positively influences satisfaction with AI writing tools.

*H2*: Perceived Ease of Use (PEOU) positively influences satisfaction with AI writing tools.

User satisfaction refers to an individual’s overall affective response and evaluative judgment based on their experience with a technology, particularly regarding whether it meets their expectations and needs (Chen et al., 2022). Satisfaction reflects a positive emotional reaction that can strengthen memory and reinforce positive engagement (Turel and Serenko, 2012). When users find their interactions with AI writing tools enjoyable and beneficial, they are more likely to use them repeatedly. Repeated engagement can foster habit formation, that is, the development of automatic use behavior. Over time, satisfying experiences with AI-assisted writing can make these tools an ingrained component of educators’ professional routines. Therefore, satisfaction with AI writing tools may play a crucial role in developing habitual use:

*H3*: Satisfaction positively influences the development of habit in the use of AI writing tools for teaching.

Habit is defined as the extent to which a behavior becomes automatic through prior learning, thereby reducing the need for conscious intention (Tamilmani et al., 2019). As habits form, individuals increasingly rely on routine actions rather than deliberate decision-making, simplifying their cognitive processes. Emotional reinforcement further strengthens these automatic behaviors, causing users to engage with AI writing tools out of habit rather than active choice (Polites and Karahanna, 2012). Once habitual, such use often becomes a default response, requiring minimal cognitive effort to continue (Wood and Rünger, 2016). Defaults exert strong influence precisely because they reduce decision-making costs (Jachimowicz et al., 2019). When the use of AI writing tools becomes habitual, the cognitive threshold for evaluating appropriateness diminishes. This reduced reflective control, reinforced by prior satisfaction and perceived efficiency, may lead educators to overuse AI writing tools, which promotes behavioral simplification by reducing the threshold for reflective control and active generation. Hence, habit may critically drive excessive use of AI writing in educational settings:

*H4*: Habit positively contributes to AI writing overuse.

### 3.2 The sequential mediation effects of satisfaction and habit

Sequential mediation (serial mediation) describes how an independent variable (IV) influences a dependent variable (DV) through a chain of mediators (Tofighi and Kelley, 2020). As discussed, favorable beliefs about AI writing tools’ usefulness and ease of use generate satisfaction, which in turn fosters habitual engagement. This mechanism aligns with Expectation-Confirmation Theory (ECT), which posits that satisfaction arises when user expectations are confirmed, promoting continued use (Rahi et al., 2021). Habit formation further aligns with dual-process models of behavior, which propose that repeated actions evolve from deliberate, reflective processes to automatic, habitual responses. Once AI-assisted writing becomes a habitual cognitive routine, decision-making and reflection are bypassed, reinforcing dependence and increasing the likelihood of overuse (Verplanken and Wood, 2006). Therefore, satisfaction and habit are expected to act as serial mediators between users’ perceptions (PU and PEOU) and AI writing overuse:

H5: PU is positively related to AI writing overuse through satisfaction and habit sequentially.

H6: PEOU is positively related to AI writing overuse through satisfaction and habit sequentially.

### 3.3 The moderating effects of Confucian values

Cultural values represent shared beliefs, norms, and priorities that shape how individuals perceive and respond to the world (Schwartz, 2012). According to the Values–Attitude–Behavior (VAB) Theory, values influence attitudes, which subsequently guide behavior (Homer and Kahle, 1988). Cultural values play a decisive role in cognitive and emotional processes, serving as the foundation for decision-making and behavioral tendencies (Zhang et al., 2019). In Chinese society, Confucian values constitute a major cultural influence. Confucianism emphasizes moral cultivation and the pursuit of social harmony (Wang, 2002). From a Confucian perspective, technology is not value-neutral, its moral worth lies in whether it contributes to harmony and collective welfare (Zhu, 2020). Thus, reliable technological engagement involves ongoing negotiation among individuals, society, and technology (Wong, 2012). The Confucian ethical view assesses whether technological practices, such as the use of AI writing tools, help individuals fulfill their professional and social responsibilities (Wong, 2012).

Accordingly, AI-assisted writing can be seen as beneficial if it helps educators fulfill their moral and pedagogical duties effectively (Zhu, 2020). Users who perceive AI writing tools as useful and easy to use are likely to develop favorable attitudes and greater satisfaction (Kashive et al., 2020). Furthermore, given that Confucian values are rooted in collectivism, individual decisions are strongly influenced by group norms and expectations (Zhang et al., 2005). Personal attitudes thus tend to align with collective consensus. Even if an individual educator initially doubts AI writing tools, they may adopt a more positive view when their peers or institutional environment endorse such practices. Consequently, under the influence of Confucian values, Chinese educators may develop positive perceptions of and reliance on AI writing tools, which may enhance satisfaction but also foster habitual use, potentially leading to overuse. Therefore, the following hypotheses are proposed:

*H7*: Confucian values positively moderate the relationship between the perceived usefulness of AI writing tools and user satisfaction.

*H8*: Confucian values positively moderate the relationship between the perceived ease of use of AI writing tools and user satisfaction.

*H9*: Confucian values positively moderate the relationship between user satisfaction with AI writing tools and the development of habitual use.

## 4 Research methodology

### 4.1 Sample and data collection

To achieve the objectives of this study, a systematic sampling procedure was implemented, as outlined below. First, the sample criteria were clearly defined in advance. The target population consisted of Chinese educators who actively use or have prior experience using AI technologies in their teaching practices. To ensure the relevance of the data, the questionnaire included a screening question asking whether respondents had used AI in their teaching. If a respondent indicated no prior use of AI, the survey was automatically terminated.

Second, a mixed-methods sampling approach was employed to enhance representativeness, utilizing both in-person intercept surveys and online distribution. In-person intercept surveys are a widely adopted method in educational research (e.g., Cheung et al., 2022; Connors and Schuelke, 2022; Stamatiadis et al., 2021) due to advantages such as higher response rates and greater control over sampling (Fink, 2003). The in-person surveys were conducted at six universities and colleges in Chongqing, an important educational center in China. To ensure institutional diversity, these universities were selected using a stratified random sampling approach based on institutional tier and type (e.g., comprehensive national universities versus applied and vocational colleges). These institutions included Chongqing University, Southwest University, Chongqing Institute of Engineering, and Chongqing City Management College. To minimize potential sampling bias during the intercept surveys, research assistants employed a systematic random approach, stationing themselves near faculty buildings and campus gates and approaching every nth (e.g., every third) passing individual identified as teaching staff during designated time blocks.

Third, to complement the in-person data collection and efficiently reach educators nationwide, an online survey was distributed via Wenjuanxing, a widely used survey platform in China. The questionnaire link was disseminated using a combination of purposive and snowball sampling strategies. Initially, the link was purposively shared within specific professional online communities, academic WeChat groups, and educational forums targeting higher education staff. A snowball element was then incorporated by encouraging these initial respondents to forward the survey link to eligible academic colleagues within their professional networks.

This study was conducted in accordance with the ethical standards for research involving human participants. Ethical approval was obtained from the relevant Institutional Ethics Review Board prior to data collection.

**Figure 1:**
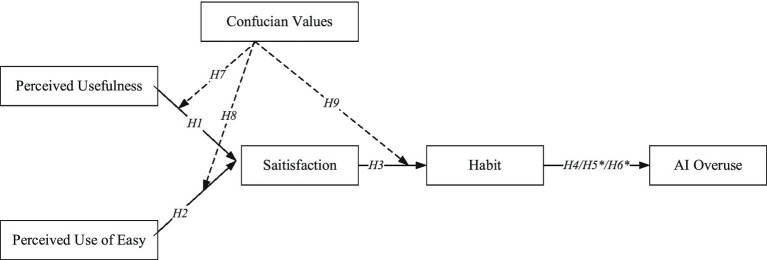
Conceptual framework.

Fourth, the survey was administered on six separate occasions to minimize common method bias and ensure more accurate sampling. The data collection period spanned from December 2024 to February 2025. A total of 486 surveys were distributed (both physically and electronically), and 471 were completed and returned, resulting in a high initial response rate of 96.91%. During the data cleaning phase, 26 responses were excluded based on predefined quality control criteria. The basis for exclusion included patterned answering behaviors (e.g., “straight-lining” or selecting the same option for all items) and unusually short completion times that fell below the minimum threshold required to read and comprehend the questions. After removing these invalid responses, a final sample of 445 valid questionnaires was retained for analysis. A brief sampling flowchart illustrating this data collection and filtration process is provided in Figure 1.

To check whether the sample size was acceptable, we followed Kock’s (2018) recommendations and used WarpPLS to calculate the minimum sample size for two powerful methods. With a statistical power of 0.80, an *R*^2^ minimum of 0.121, and a significance level of 0.05, the inverse square root approach indicated a minimum of 423 cases to be included, while the gamma-exponential method suggested a slightly smaller required number of 409. Therefore, the final sample size of 445 is sufficient for robust and reliable PLS-SEM analysis.

As shown in Table 1, the sample comprised 56.85% male and 43.15% female respondents. The majority (72.36%) were aged between 31 and 50, with no respondents under 25. Educational backgrounds were predominantly at the postgraduate level, with 72.81% holding Master’s degrees and 25.84% holding Doctorates; only 1.35% held Bachelor’s degrees. Regarding academic positions, most were Lecturers (40.67%) or Teaching Assistants (30.11%), while a smaller portion were Associate Professors (24.94%) or Professors (4.27%). In terms of AI usage frequency, 40.67% reported using AI 1–2 times per week, and 15.96% used AI more than six times per week. Teaching experience ranged widely, with the largest groups having 0–5 years (33.93%) and over 20 years (35.28%), reflecting a mix of early-career and highly experienced educators.

**Table 1:** Background information of participants.

Variable	Items	Percentage	Variable	Items	Percentage
Gender	Male	56.85	Education background	Bachelor degrees	1.35
	Female	43.15		Master degrees	72.81
Age	18 ~ 25	0.00		Doctoral degrees	25.84
	26 ~ 30	12.13	Usage frequency/per week	1 ~ 2 times	40.67
	31 ~ 40	38.20		3 ~ 4 times	26.07
	41 ~ 50	34.16		5 ~ 6 times	17.30
	51 ~ 60	13.71		6 times and above	15.96
	60 and above	1.80	Teaching experience.	0–5 years	33.93
Position	Teaching assistant	30.11		11–15 years	17.98
	Lecturer	40.67		16–20 years	8.54
	Associate professor	24.94		6–10 years	4.27
	Professor	4.27		20 years and above	35.28

### 4.2 Variables and measurement

Six main constructs were measured using established scales adapted from the existing literature. To more accurately capture participants’ self-assessments, this study employed a 7-point Likert scale ranging from “totally disagree” to “totally agree.”

The first construct, perceived usefulness (PU), was measured using four items adapted from Liesa-Orús et al. (2023), revised to fit the context of AI writing support in academic work: (PU1) “The use of AI writing support helps me complete my academic tasks more quickly”; (PU2) “The use of AI writing support improves my efficiency in completing writing and coursework tasks”; (PU3) “The use of AI writing support makes it easier for me to successfully complete academic writing tasks”; and (PU4) “I believe that AI writing support is useful in my academic work and learning activities.”

The second construct, perceived ease of use (PEOU), was assessed using three items also adapted from Liesa-Orús et al. (2023): (PEOU1) “Learning to use AI writing support tools (e.g., Qwen) would be easy for me”; (PEOU2) “It would be easy for me to make AI writing support tools perform the writing tasks I want”; and (PEOU3) “I find AI writing support tools easy to use.”

The third construct, user satisfaction (SAT), was measured with three items: (SAT1) “In general, I am satisfied with using AI writing support tools in my academic work”; (SAT2) “Compared with my expectations, using AI writing support tools is satisfying”; and (SAT3) “Compared with other methods of completing writing tasks (e.g., writing without AI support or traditional resources), I believe AI writing support tools are better.”

The fourth construct, habit (HAB), was evaluated using four items adapted from Venkatesh et al. (2012): (HAB1) “The use of AI writing support tools has become a habit for me when completing academic writing tasks”; (HAB2) “I tend to use AI writing support tools automatically when working on academic writing tasks”; (HAB3) “Using AI writing support tools has become part of my routine in completing academic work”; and (HAB4) “Using AI writing support tools feels natural to me when completing academic writing tasks.”

The fifth construct, AI overuse (OV), was measured using three items adapted from Büchi et al. (2019), with the original internet-use scale reworded to reflect AI writing support tools in academic contexts: (OV1) “I spend more time using AI writing support tools than I would like”; (OV2) “I often try to do too many academic tasks at the same time when using AI writing support tools”; and (OV3) “When I use AI writing support tools, I lose time for more important academic activities.” The adapted scale captures excessive and goal-directed reliance on AI writing tools rather than clinical addiction. To ensure content validity and clarity, the items were reviewed by experts in educational technology and behavioral research.

The sixth construct, Confucian culture (CC), was measured using six items reflecting core Confucian cultural principles: (CC1) “An individual’s behavior should be consistent with his or her social status”; (CC2) “An individual’s clothing should be appropriate for his or her social status”; (CC3) “I would not tell my family when I lose ‘mianzi’ (face)”; (CC4) “I do not like being blamed in the workplace”; (CC5) “I believe that teachers’ instructions are very important and should be followed”; and (CC6) “I mainly consider the advice of people around me when making a decision.”

Since the original questionnaire was administered in English to respondents whose first language is Chinese, a back-translation procedure was employed to ensure linguistic accuracy and conceptual equivalence.

### 4.3 Instrument validation

Prior to the full-scale data collection, a pilot study was conducted to assess the clarity, reliability, and overall suitability of the research instrument. The questionnaire first underwent a face validity review by three subject-matter experts with relevant academic and professional expertise in the research domain. Their feedback focused on the clarity, wording, relevance, and appropriateness of the measurement items. Based on their recommendations, several minor revisions were made to improve item readability and contextual suitability.

Subsequently, a pilot test involving approximately 30 educators was conducted to evaluate the comprehensibility of the questionnaire items and the internal consistency of the constructs. The pilot respondents shared similar characteristics with the target population of the main study. Feedback from the pilot participants indicated that the questionnaire items were generally clear and understandable, requiring only minor wording refinements.

Reliability analysis was performed using Cronbach’s Alpha coefficients. The results demonstrated satisfactory internal consistency for all constructs, with Cronbach’s Alpha values exceeding the recommended threshold of 0.70. Specifically, the Cronbach’s Alpha values were 0.866 for CV, 0.785 for HAB, 0.885 for AO, 0.884 for PEOU, 0.871 for PU, and 0.817 for SAT. These findings confirmed that the measurement instrument possessed acceptable reliability and was suitable for the subsequent full-scale data collection.

### 4.4 Analyses of common method variance

Common method variation (CMV) was addressed using a combination of procedural and statistical remedies. First, following the recommendations of Tourangeau et al. (2000), the survey design was carefully crafted to reduce ambiguity. Unfamiliar terms were clearly defined, vague concepts were either clarified or accompanied by examples, and questions were kept specific, simple, and concise. Additionally, the questionnaire avoided double-barreled items and complex syntax to enhance clarity during the comprehension stage of response. Second, respondent anonymity was emphasized to reduce evaluation apprehension and social desirability bias. Participants were explicitly informed that their personal information would remain confidential and would not be disclosed, in line with the guidelines proposed by Tehseen et al. (2017). Third, Harman’s single-factor test was conducted as a statistical check for CMV. The analysis revealed that the first unrotated factor accounted for only 35.416% of the total variance, well below the commonly accepted threshold of 50%. This indicates that common method variance was not a significant issue in this study.

### 4.5 Data analysis

In this study, partial least squares structural equation modeling (PLS-SEM) was used for data analysis. At present, there are two main approaches for estimating the relationship in structural equation models (Hair et al., 2019), including PLS-SEM and CB-SEM (covariance-based structural equation modeling). The two methods are complementary rather than competitive, and the choice of method depends on the objective of the study. Specifically, CB-SEM is suitable if the related theory needs to be validated (or rejected) and PLS-SEM is a better choice for theoretical development and prediction (Hair et al., 2019).

PLS-SEM is deemed suitable for this study for several compelling reasons. Firstly, it is ideal for exploratory research or theory development (Hair et al., 2021), especially in emerging fields like AI adoption influenced by Confucian values. As the research explores new territory, PLS-SEM allows for the examination of complex relationships in the data (Hair et al., 2014). The study involves multiple constructs, such as perceived usefulness, ease of use, satisfaction, habit, AI overuse, and Confucian values, as well as mediation effects, which PLS-SEM can efficiently handle due to its ability to manage intricate model structures (Hair et al., 2021). Additionally, PLS-SEM places a strong emphasis on prediction (Ghasemy et al., 2020), aligning perfectly with the study’s goal to predict educators’ satisfaction and behavioral patterns regarding AI technology use.

## 5 Results

### 5.1 Measurement model assessment

To test the measurement model, we evaluated reliability, convergent validity, and discriminant validity. Convergent validity was evaluated by checking the item loading on their respective constructs (Hair et al., 2019). The outer loading of each item ranged from 0.702 to 0.806, except for CV03 (0.596) AND HAB04 (0.471). According to Hair et al. (2014), this study analyzed the impact of above indicators deletion on internal consistency reliability because they are between 0.4 and 0.7 (Hair et al., 2014). As the deletion of above indicators increases measures above threshold, therefore, this study removed them. Reliability was tested by composite reliability (CR) and average variance extracted (AVE). As results presented in Table 2, CR values for all constructs exceeded the standard threshold of 0.7, and AVE values exceeded the cutoff values of 0.5, demonstrating sufficient construct reliability (Hair et al., 2019).

**Table 2:** The results of outer loadings, CR, AVE, and VIF.

Indicator	Measurable variable	Outer loadings	CR	AVE	VIF
CV	CV01	0.710	0.850	0.531	1.362
	CV02	0.759			1.549
	CV04	0.716			1.442
	CV05	0.712			1.528
	CV06	0.745			1.458
HAB	HAB01	0.815	0.818	0.600	1.408
	HAB02	0.747			1.244
	HAB03	0.759			1.297
AO	AO01	0.796	0.840	0.636	1.359
	AO02	0.795			1.413
	AO03	0.802			1.427
PEOU	PEOU01	0.747	0.787	0.552	1.215
	PEOU02	0.755			1.218
	PEOU03	0.727			1.160
PU	PU01	0.802	0.863	0.611	1.687
	PU02	0.785			1.636
	PU03	0.771			1.501
	PU04	0.770			1.489
SAT	SAT01	0.787	0.792	0.560	1.284
	SAT02	0.704			1.153
	SAT03	0.752			1.235

CV, Confucian values; HAB, habit; AO, AI overuse; PEOU, perceived ease of use; PU, perceived usefulness; SAT, satisfaction; CR, composite reliability; AVE, average variance extracted; VIF, variance inflation factor.

Discriminant validity was supported when the correlations among constructs were smaller than the square root of the AVE of those constructs (Bagozzi and Yi, 1988). We used the Heterotrait-Monotrait Ratio of construct correlations to confirm discriminant validity (Henseler et al., 2015). The values of correlation among constructs were all below 0.85 (see Table 3). The variance inflation factor (VIF) values were all less than 5. This indicated that multicollinearity is not a problem among the dimensions (Hair et al., 2019).

**Table 3:** The results of HTMT.

Construct	CV	HAB	AO	PEOU	PU
HAB	0.141				
AO	0.096	0.660			
PEOU	0.064	0.542	0.896		
PU	0.055	0.843	0.717	0.540	
SAT	0.152	0.617	0.893	0.827	0.692

CV, Confucian values; HAB: habit; AO, AI overuse; PEOU, perceived ease of use; PU: perceived usefulness; SAT, satisfaction.

### 5.2 Structural model assessment

**Figure 2:**
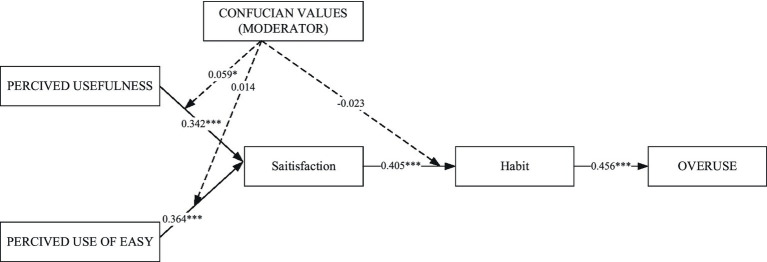
Structure model results. ***, significant at the level of 0.001; *, significant at the level of 0.1.

Following the measurement model, we assessed the structural model. The bootstrapping resampling method was used to set the repeated sampling value of 5,000 to make statistical inferences on the significance of the model coefficients. The structural model’s results are shown in Figure 2.

Table 4 presents the results of the hypothesis testing. All four proposed hypotheses were supported, with each path showing a statistically significant relationship at the *p* < 0.001 level. Specifically, PU had a significant positive influence on SAT (*β* = 0.342, *t* = 8.713, *p* < 0.001), supporting H1. Likewise, PEOU also showed a significant positive effect on SAT (*β* = 0.364, *t* = 9.149, *p* < 0.001), thus supporting H2. In addition, SAT had a strong, positive effect on HAB (*β* = 0.405, *t* = 9.416, *p* < 0.001), confirming H3. Finally, HAB significantly influenced AO (*β* = 0.456, *t* = 11.134, *p* < 0.001), providing support for H4.

**Table 4:** The results of path coefficient.

Hypothesis	Path	Coefficient	*T*-values	Support?
Direct effects				
H1	PU -> SAT	0.342	8.713***	Yes
H2	PEOU -> SAT	0.364	9.149***	Yes
H3	SAT -> HAB	0.405	9.416***	Yes
H4	HAB -> AO	0.456	11.134***	Yes
Mediating effects				
H5	PU -> SAT -> HAB -> AO	0.063	4.235***	Yes
H6	PEOU -> SAT -> HAB -> AO	0.067	5.004***	Yes
Moderating effects				
H7	CV x PU -> SAT	0.059	1.495*	Yes
H8	CV x PEOU -> SAT	0.014	0.321	NO
H9	CV x SAT -> HAB	−0.023	0.482	NO

CV, Confucian values; HAB, habit; AO, AI overuse; PEOU, perceived ease of use; PU, perceived usefulness; SAT, satisfaction. ***, significant at the level of 0.001; *, significant at the level of 0.1.

The study also examined the indirect effects to test mediation hypotheses H5 and H6. The results indicate that both PU and PEOU have significant indirect effects on AO through the sequential mediators SAT and HAB. Specifically, the indirect effect of PU on AO via SAT and HAB (H5) was significant, with a standardized coefficient of 0.063 and a t-value of 4.235 (*p* < 0.001). Similarly, the indirect effect of PEOU on AO (H6) was also significant, with a coefficient of 0.067 and a t-value of 5.004 (*p* < 0.001). These results confirm the presence of a significant sequential mediation pathway, suggesting that PU and PEOU influence overall adoption primarily through their effects on satisfaction and the development of user habits.

The moderating role of CV was examined to assess whether cultural orientation influences the strength of key relationships in the model. Among the three hypothesized moderating effects (H7 to H9), only H7 was supported. Specifically, CV significantly moderated the relationship between PU and SAT (H7), with a standardized path coefficient of 0.059 and a *t*-value of 1.495, indicating significance at the *p* < 0.10 level. This suggests that the influence of perceived usefulness on satisfaction varies depending on individuals’ level of Confucian Values. However, no significant moderating effects were found for H8 and H9. The interaction between CV and SAT on habit HAB (H9) was not significant (*β* = −0.023, *t* = 0.482, *p* > 0.05), nor was the interaction between CV and PEOU on SAT (H8), which showed a negligible and non-significant effect (*β* = 0.014, *t* = 0.321, *p* > 0.05). To clarify the nature of this interaction, a simple slope analysis was conducted (see Table 5). The relationship between PU and SAT remained positive and significant at both high (+1 SD) and low (−1 SD) levels of Confucian Values. However, the positive effect of perceived usefulness on satisfaction was notably stronger for educators with high Confucian values (*β* = 0.401, *t* = 8.92, *p* < 0.001) compared to those with low Confucian values (*β* = 0.283, *t* = 5.64, *p* < 0.001). This indicates that respondents who more strongly endorse Confucian cultural values tend to derive even greater satisfaction from AI writing tools when they perceive these tools as highly useful.

**Table 5:** Simple slope analysis for the moderating effect of Confucian values (H7).

Moderator level	Path	Beta (*β*)	T-value	*p*-value
High CV (+1 SD)	PU -> SAT	0.401	8.92	<0.001
Low CV (−1 SD)	PU -> SAT	0.283	5.64	<0.001

The structural model’s explanatory and predictive capabilities were assessed using the coefficient of determination (*R*^2^), predictive relevance (*Q*^2^), and effect size (*f*^2^). The results revealed that SAT had an *R*^2^ value of 0.358, indicating that PU and PEOU jointly explained 35.8% of the variance in satisfaction. HAB had an *R*^2^ of 0.172, showing that satisfaction accounted for 17.2% of its variance, while overall adoption (OA) had an *R*^2^ of 0.208, suggesting that habit explained 20.8% of the variance in adoption. In terms of predictive relevance, all *Q*^2^ values were above zero, confirming adequate predictive validity of the model. Specifically, SAT had a *Q*^2^ value of 0.190, HAB had 0.098, and OA had 0.130. Regarding effect sizes, PU had a medium effect on SAT (*f*^2^ = 0.154), as did PEOU (*f*^2^ = 0.175). Satisfaction had a medium effect on habit (*f*^2^ = 0.195), and habit had a medium to large effect on overall adoption (*f*^2^ = 0.262). These findings demonstrate that all model paths contribute meaningfully to explaining user behavior and system adoption.

## 6 Discussion and implications

### 6.1 Discussion

Based on the Technology Acceptance Model (TAM), this study conceptualized AI overuse in the context of AI-assisted writing and examined the moderating role of Confucian values in the process leading to overuse in educational settings. The findings provide new evidence for understanding the complex interplay between cultural values and technology use in teaching-related writing tasks.

First, this study demonstrated the sequential mediating effects of satisfaction and habit in the relationship between educators’ perceptions of AI-assisted writing tools and AI overuse. Specifically, perceived usefulness (PU) and perceived ease of use (PEOU) indirectly influenced overuse through satisfaction and the subsequent development of habitual behavior. This sequential mediation pathway clarifies how initially positive perceptions of AI-assisted writing tools, driven by their utility and usability, can ultimately contribute to excessive reliance among educators. The process begins with favorable experiences that generate user satisfaction. As educators perceive AI-assisted writing tools as satisfying, repeated engagement becomes reinforced, gradually fostering habitual use. Once habitual behavior becomes automatic and routine, it may evolve into overuse.

By empirically demonstrating this pathway from perception through satisfaction and habit to overuse, the findings substantially extend the traditional TAM, which has primarily focused on initial acceptance and adoption behavior (Musa et al., 2024). The present study shows that TAM’s core constructs and mechanisms remain relevant not only for explaining adoption but also for understanding trajectories toward excessive engagement with AI-assisted writing tools in professional educational contexts. This finding contributes to the growing literature on post-adoption technology behavior by illustrating how positive perceptions can unintentionally encourage problematic patterns of technology dependence over time.

Furthermore, the findings challenge perspectives proposed by scholars such as Bellis et al. (2021), Fasoli (2021), and Kooli et al. (2025), who predominantly conceptualize technology overuse as a form of digital addiction. Instead, the present study suggests that overuse of AI-assisted writing tools may be better understood as a consequence of productivity-oriented behavior reinforced by technological affordances. Unlike digital addiction, which is typically characterized by compulsive engagement despite negative consequences, withdrawal symptoms, and psychological dependence that overrides rational decision-making (Allcott et al., 2022), overuse in this context appears to emerge from goal-directed behavior strengthened by perceived usefulness, ease of use, satisfaction, and habitual reinforcement. Although problematic use may manifest as addiction in some situations, the current findings indicate that educators’ overuse may largely stem from task-oriented efficiency seeking rather than pathological compulsion. This distinction is theoretically important because it positions AI overuse as an unintended outcome of productivity enhancement rather than purely a psychological disorder.

Second, the findings demonstrate that cultural factors significantly shape users’ attitudes and behavioral responses toward technology (Zhao et al., 2021). In particular, Confucian values have long been recognized as influential determinants of technology-related perceptions and behaviors in Chinese society. The present findings provide empirical support for Zhu’s (2020) assertion that Confucian values influence technology perceptions and, acting as a moderator, affect both adoption and post-adoption outcomes (Zhang et al., 2019). Specifically, Confucian values significantly moderated the relationship between perceived usefulness and satisfaction, suggesting that cultural orientation plays an important role in determining how educators translate perceptions of technological utility into satisfaction with AI-assisted writing tools.

Importantly, the direction of this moderation was positive. At higher levels of Confucian values, the positive relationship between perceived usefulness and satisfaction became stronger. In other words, educators with stronger Confucian value orientations derived greater satisfaction from AI-assisted writing tools when they perceived these tools as useful. Simple slope analysis further confirmed that the relationship between perceived usefulness and satisfaction remained significant at both high (*β* = 0.401, *t* = 8.92, *p* < 0.001) and low (*β* = 0.283, *t* = 5.64, *p* < 0.001) levels of Confucian values, although the steeper slope under high Confucian values demonstrated a clear amplifying effect. Crucially, this differential pattern carries direct implications for overuse risk. Educators with higher Confucian values, deriving amplified satisfaction from useful AI tools, are more likely to progress along the satisfaction → habit → overuse pathway, as heightened satisfaction accelerates habit formation and, ultimately, excessive reliance. Conversely, educators with lower Confucian values, while still positively relating PU to satisfaction (*β* = 0.283), experience a comparatively weaker satisfaction boost from the same level of perceived usefulness. This attenuated satisfaction translates into a reduced likelihood of habitual automaticity developing, thereby positioning lower Confucian value orientations as a partial buffer against the risk of AI overuse. In short, Confucian cultural values do not merely shape how satisfied educators feel; they modulate the very magnitude of the motivational force that drives escalation from productive use toward overuse.

To understand this relationship, it is necessary to consider the core philosophical foundations of Confucianism. Collectivism, one of its central principles, emphasizes group interests over individual interests and prioritizes social harmony, fulfillment of societal roles, and contribution to collective welfare above personal autonomy (Wang and Liu, 2010). Harmony further stresses balance, relational alignment, and moral order within society (Li, 2013; Wei and Li, 2013). Collectivism explains why individuals should behave in ways that benefit society, whereas harmony explains how individuals should interact to maintain a stable and ethically responsible social environment. These values influence educators’ perceptions of technology by shaping evaluations of whether AI-assisted writing tools contribute positively to educational effectiveness, institutional goals, and broader societal responsibilities.

Consistent with this cultural perspective, the findings indicate that educators with stronger Confucian orientations experience greater satisfaction when AI-assisted writing tools are perceived as useful. Within a collectivist cultural framework emphasizing diligence, contribution, and harmonious functioning, AI writing tools may be viewed as valuable resources for improving teaching efficiency, supporting student learning, and facilitating smoother educational processes. Consequently, when educators perceive these tools as useful for fulfilling professional and social responsibilities, satisfaction is amplified because technology use aligns closely with culturally valued goals and ethical expectations.

At the same time, it is important to acknowledge that efficiency-seeking behavior is not unique to collectivist cultures. Individualistic cultures, which emphasize personal achievement, autonomy, and competitiveness, may similarly encourage strong perceptions of usefulness and intensive engagement with AI technologies. In such contexts, educators may perceive AI-assisted writing tools as valuable for maximizing personal productivity, reducing workload, or enhancing career performance. Thus, while the present findings demonstrate that Confucian collectivism amplifies satisfaction derived from useful AI-assisted writing tools by aligning technology use with collective ethical goals, the broader pathway toward habitual use and potential overuse may emerge across cultures through different motivational rationales. This finding complements prior research on AI effectiveness in education (e.g., Chou et al., 2022; Zheng et al., 2023) by introducing an important cultural dimension that explains why technology effectiveness may be interpreted differently across cultural environments.

However, the hypothesis proposing that Confucian values would strengthen the relationship between perceived ease of use and satisfaction was not supported. One possible explanation is that perceived ease of use represents a largely functional and cognitive assessment focused on the effort required to operate and integrate technology into daily tasks (Davis, 1989). In contrast, Confucian values primarily emphasize ethical conduct, collective welfare, and social responsibility (Wang and Liu, 2010). These values are more likely to shape evaluations concerning whether technology is beneficial or meaningful rather than whether it is easy to use. The absence of moderation between perceived ease of use and satisfaction therefore suggests that usability evaluation reflects a relatively universal human–computer interaction mechanism driven primarily by cognitive load and operational simplicity. Because judgments regarding ease of use are fundamentally utilitarian and efficiency-oriented, they may remain relatively stable across cultural contexts and less influenced by deeper cultural value systems.

Similarly, the hypothesis proposing that Confucian values would moderate the relationship between satisfaction and habit was not supported. Satisfaction itself appears to function as the primary driver of repeated behavior by providing immediate positive reinforcement. When educators experience rewarding outcomes from using AI-assisted writing tools, they are naturally more likely to repeat the behavior, gradually reinforcing habit formation. Habit formation is characterized by increasing behavioral automaticity (Bermúdez and Felletti, 2021) and operates largely through universal psychological reinforcement mechanisms (Skinner, 1965). As repeated behavior becomes increasingly automatic, the influence of higher-order cultural values may diminish. Consequently, although Confucian values strongly shape the earlier evaluative stages of technology engagement by influencing perceived usefulness and satisfaction, the subsequent transition from satisfaction to habitual behavior appears to follow a more universal reward-driven process. This finding challenges assumptions that cultural moderation necessarily extends across the entire post-adoption process (e.g., Nikkhah et al., 2025; Tarhini et al., 2017) and instead suggests that cultural influence may weaken during later automatic stages of technology use.

### 6.2 Theoretical implications

This study offers several theoretical contributions. First, this study extends the traditional application of the Technology Acceptance Model by demonstrating its relevance beyond initial acceptance and adoption, offering insight into the phenomenon of technology overuse. By showing how core TAM constructs, PU and PEOU, sequentially influence overuse through satisfaction and habit, the findings expand TAM’s explanatory power to include later-stage and potentially problematic technology engagement. This contributes to the evolution of theoretical models that account for the full continuum of technology use, from initial adoption to excessive utilization.

Second, this study refines how cultural values, particularly Confucian values, interact with users’ psychological processes in technology adoption. The findings show that Confucian values significantly moderate the relationship between perceived usefulness and satisfaction, but not between perceived ease of use and satisfaction. This distinction highlights that cultural values exert a stronger influence on value-oriented evaluations, such as the perceived contribution of technology to collective goals or ethical standards, than on functional or usability-based perceptions. These results suggest that cultural context does not uniformly shape all aspects of technology engagement; instead, its impact is selective and context-dependent. By clarifying where cultural influence is most pronounced, the study challenges broad theoretical claims about the universal moderating power of culture and calls for more targeted investigations that account for both cultural and cognitive dimensions of technology use.

Third, the study contributes to ongoing theoretical debates by reframing technology overuse not as a form of addiction, but as a consequence of productivity-driven behaviors reinforced by technological features. The evidence supports a model in which overuse emerges through satisfaction and habitual use, suggesting that such behavior may stem from goal-directed engagement that becomes excessive under certain contextual pressures. This theoretical lens offers an alternative to addiction-based frameworks, emphasizing the role of psychological reinforcement and environmental demands rather than solely pathological dependency.

Finally, the findings highlight the theoretical importance of incorporating cultural context into models of technology use and overuse, especially in non-Western settings. By empirically demonstrating that Confucian values interact with key psychological constructs, such as perceived usefulness and satisfaction, the study underscores that cultural factors are not passive background variables but active components shaping user engagement. This reinforces the need for culturally responsive theories that better reflect the diversity of global technology experiences.

It is also important to acknowledge that efficiency-driven AI overuse is not unique to Confucian collectivist cultures. In individualist, capitalist societies, employees and educators face comparable pressures to work faster and produce more, driven by personal advancement, salary incentives, and competitive performance metrics (Büchi et al., 2019; Vanden Abeele, 2021). While the motivational pathway differs,collectivist cultures are driven by fulfilling social roles and maintaining group harmony, whereas individualist cultures are driven by personal achievement and self-interest, the behavioral outcome of AI overuse may be similar across cultural contexts (Nikkhah et al., 2025). Future comparative research, such as cross-national studies contrasting China and Western countries, is needed to empirically test whether the sequential mediation pathway (PU/PEOU → Satisfaction → Habit → Overuse) operates similarly across cultural settings.

### 6.3 Practical implications

Based on the study’s findings, several practical implications emerge for guiding the use of AI technologies in educational settings, particularly in promoting sustainable, high-quality use and fostering reflective engagement in contexts shaped by Confucian cultural values. First, the prominent role of perceived usefulness, moderated by cultural norms, underscores the need for educational policymakers to support the design and implementation of AI tools and related training programs that align with educators’ professional identities and collective cultural values. For example, AI writing tools should be developed to enhance student learning outcomes, encourage collaborative learning, and support social harmony, principles central to Confucian educational ideals. Training programs should emphasize these values to increase educator satisfaction, foster responsible technology use, and promote reflective engagement with AI tools.

Secondly, this study reframes AI overuse as a consequence of productivity-driven, goal-directed behaviors, specifically, the progression from perceived usefulness and ease of use to satisfaction and habit, rather than personal addiction or lack of self-control. While the present investigation did not directly measure systemic factors such as work overload, existing literature suggests that such external institutional demands often drive the initial hunt for technological efficiency. Therefore, educational institutions should be mindful that heavy reliance on AI writing tools may be symptomatic of underlying workload pressures. Addressing these broader institutional demands, rather than merely restricting technology use, may be a more effective strategy for promoting healthy digital engagement.

Thirdly, the strong relationship between satisfaction and habit formation highlights the importance of ensuring that satisfaction with AI use is rooted in meaningful professional benefits for the educators themselves, rather than in mere convenience. Because this study specifically investigates educators’ use of AI writing tools, educational policy should prioritize the selection and implementation of AI tools that support teachers in their daily work. This involves integrating systems that effectively reduce administrative burdens and streamline routine drafting tasks, thereby freeing educators to focus on pedagogical innovation and complex interactions with students. Crucially, institutions must ensure that the habitual use of these tools does not erode teachers’ professional agency; educators must retain their critical evaluative skills and active role in curriculum design, rather than passively delegating these responsibilities to AI. Furthermore, government support for the development of ethical AI systems, including features that promote healthy usage patterns and user self-regulation, can help prevent excessive dependence and ensure AI remains a supportive tool for educators’ professional practice.

### 6.4 Limitations and future research

Despite its contributions, this study has several limitations that offer valuable directions for future research. Firstly, the study was conducted within a specific cultural context, focusing on educators who are influenced by Confucian values. While this cultural focus enabled a deeper exploration of how value systems shape technology use, it also limits the generalizability of the findings. Educators from different cultural backgrounds, as well as other user groups such as students or professionals in non-educational sectors, may have different experiences and motivations when engaging with AI technologies. Future research should replicate this study across a broader range of cultural and professional contexts to determine whether the relationships identified in this model are culturally specific or more universally applicable.

Secondly, the study likely employed a cross-sectional design that captures data at a single point in time. Although this approach is effective for identifying associations among variables, it does not allow for conclusions about causality or for understanding the development of technology-related behaviors over time. Longitudinal studies are recommended to track how educators’ perceptions, satisfaction, habits, and tendencies toward overuse evolve. Such research would provide stronger evidence for the proposed sequential mediation process and help clarify the dynamic influence of cultural values during different stages of technology engagement.

Thirdly, the reliance on self-reported data presents another limitation. Although widely used, self-report measures can be influenced by social desirability bias, memory inaccuracies, and subjective interpretations of survey items. Future research could adopt mixed-method approaches that incorporate interviews, focus groups, or observations to provide deeper insights into user experiences. Where appropriate and ethical, objective behavioral data such as system usage logs could also be used to complement self-report data and enhance the accuracy of findings.

Fourthly, the study hypothesized that Confucian values would moderate the relationship between satisfaction and habit. However, the results did not support this hypothesis. The findings indicate that satisfaction, rather than cultural values, is the more decisive factor in predicting whether users will continue engaging in AI use as a habitual behavior. This lack of a moderating effect suggests that cultural influences may not uniformly affect all stages of technology use. Future research could address this limitation by exploring alternative cultural frameworks or employing more nuanced instruments for measuring cultural values. It may also be beneficial to conduct subgroup analyses or comparative studies across cultures to further examine when and how cultural values influence technology-related behaviors.

## Data Availability

The original contributions presented in the study are included in the article/supplementary material, further inquiries can be directed to the corresponding author/s.
